# Hydrodynamic and Performance Evaluation of a Porous Ceramic Membrane Module Used on the Water–Oil Separation Process: An Investigation by CFD

**DOI:** 10.3390/membranes11020121

**Published:** 2021-02-08

**Authors:** Guilherme L. Oliveira Neto, Nívea G. N. Oliveira, João M. P. Q. Delgado, Lucas P. C. Nascimento, Hortência L. F. Magalhães, Paloma L. de Oliveira, Ricardo S. Gomez, Severino R. Farias Neto, Antonio G. B. Lima

**Affiliations:** 1 Federal Institute of Education Science and Technology of Piauí, Floriano 64808-475, Brazil; guilherme@ifpi.edu.br; 2 Technical School of Floriano, Federal University of Piauí, Floriano 64808-605, Brazil; niveagomes@ufpi.edu.br; 3 CONSTRUCT-LFC, Department of Civil Engineering, Faculty of Engineering, University of Porto, Porto 4200-465, Portugal; jdelgado@fe.up.pt; 4 Department of Mechanical Engineering, Federal University of Campina Grande, Campina Grande 58429-900, Brazil; lucaspereira.cn@hotmail.com (L.P.C.N.); ricardosoaresgomez@gmail.com (R.S.G.); antonio.gilson@ufcg.edu.br (A.G.B.L.); 5 Department of Chemical Engineering, Federal University of Campina Grande, Campina Grande 58429-900, Brazil; palomalima_eq@yahoo.com (P.L.d.O.); severino.rodrigues@professor.ufcg.edu.br (S.R.F.N.)

**Keywords:** produced water, membranes, separation, CFD, Ansys Fluent

## Abstract

Wastewater from the oil industry can be considered a dangerous contaminant for the environment and needs to be treated before disposal or re-use. Currently, membrane separation is one of the most used technologies for the treatment of produced water. Therefore, the present work aims to study the process of separating oily water in a module equipped with a ceramic membrane, based on the Eulerian–Eulerian approach and the Shear-Stress Transport (SST k-ω) turbulence model, using the Ansys Fluent^®^ 15.0. The hydrodynamic behavior of the water/oil mixture in the filtration module was evaluated under different conditions of the mass flow rate of the fluid mixture and oil concentration at the entrance, the diameter of the oil particles, and membrane permeability and porosity. It was found that an increase in the feed mass flow rate from 0.5 to 1.5 kg/s significantly influenced transmembrane pressure, that varied from 33.00 to 221.32 kPa. Besides, it was observed that the particle diameter and porosity of the membranes did not influence the performance of the filtration module; it was also verified that increasing the permeability of the membranes, from 3 × 10^−15^ to 3 × 10^−13^ m^2^, caused transmembrane pressure reduction of 22.77%. The greater the average oil concentration at the permeate (from 0.021 to 0.037 kg/m^3^) and concentrate (from 1.00 to 1.154 kg/m^3^) outlets, the higher the average flow rate of oil at the permeate outlets. These results showed that the filter separator has good potential for water/oil separation.

## 1. Introduction

The water produced is a mixture of different materials (fluids, dissolved and suspended solids) originating from the extraction of oil and gas in underground reservoirs. During oil production, a large amount of water is produced with a high load of organic and inorganic compounds, featuring extremely saline, oily, and toxic effluents for living beings. In general, for each barrel of oil produced, about three barrels of residual water are released [1,2,3].

Due to the high risk to health and environmental imbalance [2,4], Brazilian and international environmental regulatory companies have established norms and regulations related to the mandatory treatment of this produced water before its disposal or reuse [3]. In Brazil, the National Environment Council (CONAMA) is the main regulator of effluent release conditions and standards, establishing a maximum oil and wax content of 20 mg/L for disposal [5].

Currently, there are different technologies and methods for treating water polluted with oil, which takes into account the physical, physical–chemical, chemical, and biological principles that occur in the processes [4,6]. Among these methods are flotation [7,8,9], coagulation [6,10,11,12,13], the use of hydrocyclones [14,15,16,17], biological treatment [18,19,20,21] and membrane separation technologies [21,22,23].

In general, the higher the crude oil content in the water, the lower the removal efficiency; therefore, it is necessary to use a combination of different methods that can enhance the processes of water purification [10]. Khan et al. [12], for example, combined coagulation and flocculation to remove large particles of oil and inorganic matter from the produced water and, as a result, obtained a favorable environment for the biodegradation microorganisms, and finally, using microfiltration membrane removed the remaining dissolved oil microdroplets.

According to Jepsen et al. [14], traditional technologies for the treatment of produced water (such as the use of hydrocyclones) are already working within their fundamental limit, so new filtration technologies must be proposed to enhance water purification. For example, hydrocyclone is not efficient enough to treat oil dispersed in water with oil droplets of small diameter, especially when the oil concentration is low.

In this context, ceramic membrane technologies have excelled in presenting excellent chemical, thermal and mechanical properties, favoring high performance in severe operating conditions, such as high temperatures and the presence of aggressive chemicals [20]. Another advantage of membrane technology is related to the large volume of treated water, thereby, membrane separation has become more efficient compared to conventional techniques [24].

Membrane separation processes can be classified into four categories according to the pore size: microfiltration, ultrafiltration, nanofiltration, and reverse osmosis [24,25,26]. The pore size of the membrane determines the filtration properties of water/oil emulsions, which can also vary, depending on the concentration of the oil in the emulsion and, consequently, on the size of the oil drop [13,27].

The operational parameters also play an important role in the potential of membrane separation. For example, the velocity of the feed flow [28], the transmembrane pressure [29], the temperature, the pH, and the size of the dissolved molecules [24] are very important during oily wastewater separation processes.

The use of ceramic membranes to treat oily effluents has grown considerably due to their high filtration efficiency, excellent hydrophilic properties, and chemical and hydrothermal stability [30]. However, the performance is strongly affected by the concentration polarization phenomenon (formation of an oil layer and other contaminants from the effluent parallel to the membrane surface) [31,32,33,34] and, by the membrane encrustation that generally occurs as a result of adsorption of components of the feed solution on the membrane surface [35,36].

All potential assigned to the ceramic membranes justifies the intensification of scientific works that seek to enhance the treatment of produced water with this technology [29,31,37,38,39,40]. In the literature, several studies have been reported using computational fluid dynamics as a tool to investigate the process separation of oily waters [40,41,42,43,44,45,46].

Cunha (2014) [39] numerically studied the treatment of petroleum industrial effluents using a ceramic membrane. In this work, a mathematical model, later implemented in the Ansys CFX, was developed to describe the physical problem, aiming to evaluate the formation of the concentration polarization layer on the porous membrane surface. The author observed that the formation of the polarization layer by concentration is influenced by the hydrodynamic behavior of the flow and by the properties of the fluid mixture and the porous medium.

Magalhães et al. [45] in their work on the non-isothermal treatment of oily waters, investigated the thermal influence on the oil/water separation process using a module equipped with a ceramic membrane. The authors observed that an increase in the temperature has influenced the fluid properties and the flow behavior inside the equipment, causing changes in the pressure, concentration, and speed fields.

Shirazian et al. [41] investigate the mass transfer in wastewater treatment using membrane reactors. The authors observed that the velocity reached its full development after a short distance from the reactor inlet, and that the total flow of contaminant decreased dramatically in the region close to the membrane entrance, due to the resistance imposed by the membrane to the flow passage.

In this context, the CFD (Computational Fluid Dynamics) technique provides information and new models for the application of membranes and contributes to the understanding of the filtration mechanisms [43,46,47,48,49,50,51,52,53,54]. The computational fluid dynamics enables the understanding and development of new membrane separation processes, allowing researchers to study, in detail, phenomena such as fouling, concentration polarization, and pore-clogging, responsible for reducing the filtration rate and favoring contamination of the permeate.

Based on the CFD simulations, Yang et al. [53] have studied a new configuration of a cylindrical 37-channel porous inorganic membrane tube by increasing membrane filtration area and increasing permeation efficiency of inner channels. In this very interesting paper, the authors have concluded that the permeate efficiency of the inner channels is smaller than that of the outer channels and that the difference in permeate flux of a channel in different radial rings diminishes gradually as the resistance of the skin layer increases. Tong et al. [54] reported a similar CFD study using a 19-core tandem ceramic membrane module. The authors concluded that when the volume flow rate changes from 26 to 89 m^3^/h, the resistance of each part of the membrane module system increases gradually. The increase in resistance loss in the membrane element is faster than that in the plates and the bell mouths.

Because of the above, this work aims to study the hydrodynamics of water and oil flow in a ceramic membrane module, and verify the effect of the separation process variables on the equipment’s performance via computational fluid dynamics (Ansys Fluent^®^).

## 2. Methodology

### 2.1. The Physical Problem and Geometry

**Figure 1:**
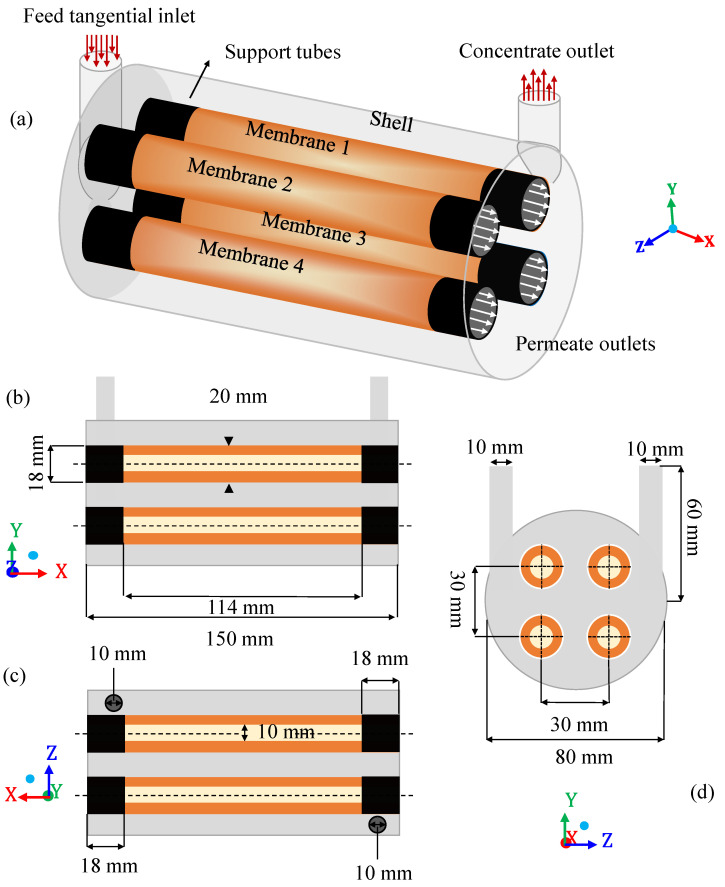
Scheme of the geometry of the separation module and its dimensions (**a**) Permeation module, (**b**,**c**) Longitudinal views of the permeation module, (**d**) Frontal view of the permeation module.

In this study, the flow of a mixture of oil and water in a porous membrane system was analyzed. It is, therefore, a two-phase system with fluid flow in porous and non-porous media. The ceramic membrane filtration module is of the shell-and-tube type (domain understudy), consisting of the main cylinder (hull) with a tangential inlet and outlet and four internal cylindrical tubes which are the porous ceramic membranes (membranes 1, 2, 3, and 4), as shown in Figure 1. Table 1 summarizes all the geometric dimensions of the separation module used in the numerical simulations.

The tangential inlet of the module is a tube of a circular cross-section through which the contaminated effluent enters the system. The tangential outlet tube, also of circular cross-section, is intended for the outlet of the concentrate. Part of the filtrate (oil) is retained inside the membranes after filtration. Thus, the separation between oil and water occurs as the feed mixture, containing a certain oil concentration, enters tangentially into the separator. In this way, the fluid mixture is forced to cross the membranes, in such a way that the oil fraction is retained in the porous structure, and only the aqueous phase easily crosses the membrane, generating the permeate. This is caused, mainly due to the difference in transmembrane pressure. Finally, the four outlets coming from the membranes receive the permeate flow, while the outlet in the hull receives the concentrate (mixture with high oil concentration).

For a numerical solution to the conservation equations that govern the domain under study, it is necessary to convert the continuous domain into a discrete domain. These control volumes are three-dimensional partitions of the geometric domain, composed of edges and nodal points, in which the discretized governing equations must be solved, and represent the physical phenomenon. The numerical method causes errors of truncation and idealizations, which decrease, as elements are added to the mesh, that is, as the finite limits of the solution are reduced.

From a computational point of view, the separation module has nine structural domains, which are: the four volumes of fluids related to the four membranes (porous medium, through which water flows and the oil is retained), the four permeates (part membrane, through which the permeate flows) and the cylindrical hull, comprised between the internal surfaces, which limit the separator and the outer surfaces of the membranes (non-porous medium, through which the mixture of oil and water flows towards the outlet). From this, to adequately predict the proposed problem, three hybrid meshes (tetrahedral and hexahedral elements) with different densities of control volumes (elements) were built, using the Ansys Meshing^®^ software. To build the hull domain, hexahedral elements were used near the outer walls of the membranes and the hull itself, and tetrahedral elements for the other regions. To describe the domain of the membranes, structured meshes were built with only hexahedral elements, which was maintained for the cylindrical domains (inner tube of the membrane through which the permeate flows). The entire domain was developed using the mesh construction technique called o-grid.

After building the meshes using the Ansys Meshing^®^ software, it was observed that all meshes were within the recommended limits for deformation (below 0.95) and orthogonality (above 0.1) values.

**Figure 2:**
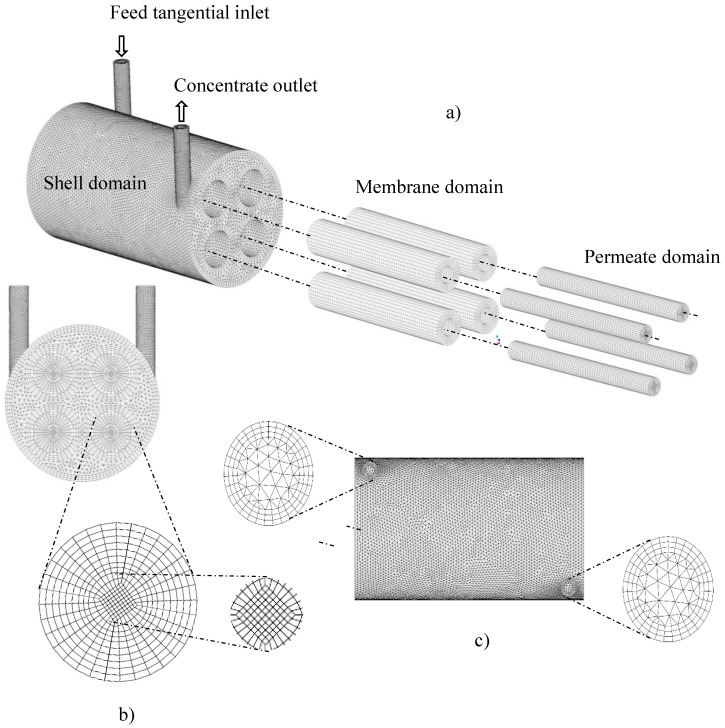
The numerical mesh of the module under study, (**a**) Detail of the membranes, (**b**) Details at the exit of the membrane, and (**c**) Details of the inlet and outlet of the permeation module.

Figure 2 shows mesh 2, with emphasis on the studied domains. In this figure, the mesh of the hull, membrane, and permeate domains can be seen. Besides, it is possible to see a cross-section of the entire module, focusing on the membrane mesh, as well as the mesh entrance and exit regions.

After construction, these meshes were evaluated for dependence on the numerical results obtained by the simulations with the number of elements.

### 2.2. Mathematical Modeling

To describe the flow of fluids in the regions of the hull and cylindrical tube (through which the permeate flows), the multiphase model with the Eulerian–Eulerian type formulation was used. This method of approach is capable of modeling multiple phases, treated as separate, but interacting with each other. The phases can be liquid, gaseous, or solid, in almost any combination. Eulerian–Eulerian solution treatment is used for each phase separately, even if one of the phases is made up of particles. The model makes no distinction between the fluid–fluid and fluid–solid (granular) flow. A granular flow is simply one that involves at least one phase designated as granular. A single pressure is shared by all phases, and the equations of moment and continuity are solved for each phase [55].

The formulation of the model is based on the assumption that two or more phases are continuous and immiscible. For each phase added to the mathematical model, a new variable corresponding to the volumetric fraction (α_q_), of the respective phase “q”, is introduced. The volumetric fraction represents a relationship between the volume occupied by each phase and the total volume of the cell. In each cell, the laws of conservation of mass and linear moment are satisfied for each phase individually.

In each control volume (V), the sum of the volumetric fractions of the phases is equal to 1 (one). So, you can write:

(1) $${\;V}_{q}=\overset{}{\underset{V}{\int }}\alpha_{q}\mathrm{dV},\;$$

where:

(2) $$\overset{n}{\underset{q=1}{\sum }}\alpha_{q}=1.$$

The effective density of phase q is defined as follows:

(3) $${\hat{\rho }}_{q}=\alpha_{q}\rho_{q},$$

where $\rho_{q}$ is the physical density of phase q.

Based on this methodology, the following conditions can be met:

$\alpha_{q}=1$: indicates that the cell volume is filled by the q phase.

$\alpha_{q}=0$: indicates that the cell volume does not contain the q phase.

$0<\alpha_{q}<1$: indicates that the cell volume is partially filled by the q phase. In this condition, there is an interface between the q phase and one or more phases present in the cell.

Based on the local value of $\alpha_{q}$, of the n phases existing in the physical process, the properties and process variables are weighted in each region of the multiphase flow. In this work, it was considered that the mass conservation and linear momentum equations are solved for each of the present phases (continuous and dispersed). For each of the physical situations, the following considerations were adopted:

- Flow in permanent and isothermal regime;
- Newtonian fluid, incompressible and with constant thermo-physical–chemical properties;
- Interfacial mass transfer, interfacial momentum, and mass source are negligible;
- The non-drag interfacial forces (lift forces, wall lubrication, virtual mass, turbulent dispersion, and solid pressure) are neglected;
- The walls of the separation module are rigid (not deformable) and have null roughness;
- The fluid flow in the feed is considered as a mixture of immiscible water and oil (not emulsion);
- The porous medium (ceramic membrane) has a uniform distribution of porosity and permeability;
- There are no reaction or adsorption phenomena of the solute on the contact surface in the porous medium.

#### 2.2.1. Formulation for the Non-Porous Domain

The mass conservation equation for multiphase flow is given by Equation (4), as follows:

(4) $$\nabla .\left(\alpha_{q}\rho_{q}{\vec{v}}_{q}\right)=0.$$

where the sub-index “q” represents the phase involved in the water/oil two-phase mixture; α, ρ and $\vec{v}$ are the volumetric fraction, density, and velocity vector, respectively.

The linear momentum conservation equation for multiphase flow is defined by Equation (5), as follows:

(5) $$\nabla .\left(\alpha_{q}\rho_{q}{\vec{v}}_{q}{\vec{v}}_{q}\right)=-\alpha_{q}\nabla P+\nabla \cdot {\overset{=}{\tau }}_{q}+\alpha_{q}\rho_{q}\vec{g}+\overset{n}{\underset{p=1}{\sum }}{\vec{R}}_{\mathrm{pq}}.\;$$

where ${\overset{=}{\tau }}_{q}$ is the stress tensor for the q phase and ${\vec{R}}_{\mathrm{pq}}$ is the term for the interface forces between phases p and q, P is the pressure, shared by all existing phases.

The tension related to the phase can be defined by:

(6) $${\overset{=}{\tau }}_{q}=\alpha_{q}\mu_{q}\left(\nabla {\vec{v}}_{q}+\nabla {\vec{v}}_{q}^{T}\right)+\alpha_{q}\left(\lambda_{q}-\frac{2}{3}\mu_{q}\right)\nabla .{\vec{v}}_{q}\overset{=}{I},$$

where $\mu_{q}$ and $\lambda_{q}$ are, respectively, the viscosity and shear stress of phase q, and $\overset{=}{I}$ is the unit tensor.

The interface strength depends on friction, pressure, cohesion, and other effects, such that the following condition must be met:

(7) $${\vec{R}}_{\mathrm{pq}}\;={\vec{\;R}}_{\mathrm{qp}}\;\mathrm{and}\;{\vec{R}}_{\mathrm{qq}}=0.$$

The Fluent solver uses a simplified interaction term, as follows:

(8) $$\overset{n}{\underset{p=1}{\sum }}{\vec{R}}_{\mathrm{pq}}=\overset{n}{\underset{p=1}{\sum }}K_{\mathrm{pq}}\left({\vec{v}}_{p}-{\vec{v}}_{q}\right),\;$$

where $K_{\mathrm{pq}}=K_{\mathrm{qp}}$ is the interfacial momentum transfer coefficient; ${\vec{v}}_{p}$ and ${\vec{v}}_{q}$ are the velocities of the phases.

For a two-phase flow, it is assumed that the second phase is in the form of drops. This has an impact on how each fluid is assigned to each phase, for example, in flows where there are unequal quantities of two fluids, the predominant fluid must be modeled as the primary fluid since the dispersed fluid is more likely to form droplets or bubbles [56]. The exchange coefficient for bubbling, liquid–liquid, or gas–liquid mixtures can be written as follows:

(9) $$K_{\mathrm{pq}}=\frac{\rho_{p}f}{6\tau_{p}}d_{p}A_{i},\;$$

where $A_{i}$ is the interfacial area defined by $A_{i}=\frac{6\alpha_{p}\left(1-\alpha_{p}\right)}{d_{p}},\;f$ is the drag function, which is defined according to the exchange rate model used, and the term $\tau_{p}$ is the “particulate relaxation time”, defined as follows:

(10) $$\tau_{p}=\frac{\rho_{p}d_{p}{}^{2}}{18\mu_{q}},\;$$

where $d_{p}$ is the diameter of the drop.

To determine the drag function f, the Schiller and Naumann model [57] was used, as follows:

(11) f=CDRe24,

where $C_{D}$, is the drag coefficient, given by:

(12) CD={24(1+0.15Re0,687)/Re, Re≤1000 0.44, Re>1000,

with Re being the relative Reynolds number, defined for the primary phase q and the secondary phase p, as follows:

(13) Re=ρp|v→p−v→q|dpμq. 

To describe the flow of the mixture inside the domain in the turbulent regime, we used the Shear-Stress Transport (SST k-ω) model developed by Menter [58]. This model is based on the coupling of the standard k-ω models [59] with the k-ε model [60], which, respectively, are characteristic for presenting good results near the walls and in the regions away from the numerical domain wall. This model was applied individually for each continuous and dispersed phases. Details about of the turbulence model used in this research can found in the literature [52].

#### 2.2.2. Formulation for Porous Medium

In the Eulerian–Eulerian multiphase model, the general approach of modeling porous media, physical laws, and equations is applied to the corresponding phase for the conservation of mass and linear momentum. In Fluent software, a porous medium is modeled as a region containing fluid elements, where the equation for the linear momentum is modified by the addition of a source term of dissipation. This source term is composed of two parts: one referring to the loss of viscous effects (Darcy’s Law, the first term on the right side of Equation (14)) and one referring to the loss of inertial effects (the second term on the right side of Equation (14)).

(14) $$S_{i}=-\left(\overset{3}{\underset{j=1}{\sum }}M_{\mathrm{ij}}{\mu v}_{j}+\overset{3}{\underset{j=1}{\sum }}N_{\mathrm{ij}}\frac{1}{2}\rho \left|v\right|v_{j}\right),\;$$

where $S_{i}$ is the source term for the i-th (x, y, or z) equation of the linear momentum, |v| is the magnitude of the velocity, and M and N are prescribed matrices. This momentary sink contributes to the pressure gradient in the porous cell, creating a pressure drop that is proportional to the speed of the fluid in the cell.

Thus, homogeneous porous media are defined as follows:

(15) $$S_{i}=-\left(\frac{\mu }{K_{i}}v_{i}+C_{2}\frac{1}{2}\rho \left|v\right|v_{i}\right),$$

where $K_{i}$ is the permeability, and $C_{2}$ is the inertial resistance factor, simplifying the matrices M and N as diagonal matrices, with $\frac{1}{K_{i}}$ and $C_{2}$, respectively, occupying the values on the matrix diagonals. Given the low velocities developed in the volumes relative to the porous medium, the term referring to inertial resistance was neglected in this research.

### 2.3. Boundary Conditions

(a) Module input

At the input of the separator module, a prescribed mass flow condition has been established. Specifying the mass flow rate allows the total pressure to vary in response to the numerical solution. In this boundary condition, the absolute reference system, the flow direction (normal to the inlet surface), the turbulence intensity, I = 5%, and the turbulent viscosity ratio $R_{\mu }$ = 10, given by Equations (16) and (17), have been established.

(16) $$I\equiv \frac{u^{\prime }}{\bar{u}}$$

and

(17) $$R=\frac{\mu_{t}}{\mu },$$

where $u^{\prime }$ is the rate of velocity fluctuation, and $\bar{u}$ is the mean velocity of free flow. The values of k and ε are computed as a function of this intensity [60].

(b) Concentrate and permeate outputs

For the permeate and concentrate outputs, a prescribed pressure boundary condition was applied. There was a zero gauge pressure at the outlets, that is, the ambient pressure. The pressure difference between the input of the separation module and the outputs (atmospheric pressure) drives the flow of produced water through the separation module.

(c) Module wall and membrane surface

The conditions of the wall were used to connect the fluid and solid regions, surfaces of the module, and membranes, in contact, externally, with the volume of the hull and, internally, with the domains related to permeate.

On the internal surfaces of the hull, surfaces of the supports (membrane ends), hull and permeate, non-slip boundary conditions, and null roughness were applied. For the internal and external surfaces of the membranes, the condition of the interior wall (open surface) was used, which allowed the flow of produced water through the membranes. Additional information can be found in Table 2.

### 2.4. Numerical Procedures

For the pressure–velocity coupling, the Coupled algorithm, available in the Ansys Fluent^®^ software, was used. The Coupled algorithm solves the continuity equation based on the results of calculating the conservation of linear momentum and pressure, in a coupled way, which gives it, concerning the segregated solution algorithms, a convergence range with a smaller number of iterations.

The relaxation factors used in the pressure–velocity coupling methods called Coupled are shown in Table 3. The convergence criteria used in the simulations are described in Table 4.

### 2.5. Thermo-Physical Properties of Fluids

The physical properties of the substances used in the numerical simulations are described in Table 5.

### 2.6. Simulated Cases

In this research, different simulations were performed, varying the mass flow, $\dot{m}$, the volumetric oil concentration at the entrance, $C_{0}$, the average diameter of the oil droplet particles, $d_{p}$, the permeability of the membranes, K, and the porosity of the membranes, ε. The idea is to evaluate the effect of these variables on the fluid dynamic behavior of the phases inside the module of porous ceramic membranes and the separation performance of this equipment. The values of the variables at entry in the initial conditions, in each case studied, are shown in Table 6.

Case 5 was considered as a standard case. With Cases 1, 2, and 3, the influence of the mesh on the results obtained was investigated. Cases 2, 4, and 5 were simulated to verify the effect of the mass flow rate of the mixture at the entrance of the separation module, and Cases 5, 6 and 7, were used to evaluate the effect of the oil volumetric concentration at the entrance of the separation module. The effect of the average diameter of the oil droplet was verified with the results obtained with the simulations of Cases 5, 8, and 9, and the effect of the membrane permeability, with Cases 5, 10, and 11. To verify the influence of the porosity of the membranes in the process, Cases 5, 12, and 13 were simulated.

## 3. Results

### 3.1. Mesh Refining Analysis

The computational mesh generation stage is one of the most important to obtain more accurate results in a computer simulation. An inadequate mesh, which may be in terms of the number and type of elements, can make CFD simulation unfeasible, or generate very negative results, especially concerning the solution’s precision, the required simulation time, and the convergence rate (or divergence) of the results.

So, to achieve coherent results in CFD, it is necessary to carry out a mesh study, from which a comparison is made between the results obtained with a more refined mesh, with results from others, less refined, so that the results become independent of the mesh (concerning the number of elements). For that, a study of the effect of the mesh was made using the results obtained with Cases 1, 2, and 3 of Table 6.

Table 7 summarizes the results obtained with the cases studied for the mesh independence test. These results are presented in terms of the following parameters: transmembrane pressure (∆P); average oil concentration at the permeate outlets (AOCP); average oil concentration in the concentrate (AOCC); average oil mass flowrate at the permeate outlets (AOMP); average speed of the mixing at the permeate outlets (AVMP); the average volume of oil in the hull (AVOH); the average volume of oil in the permeate (AVOP) and the average volume of oil in the membranes (AVOM).

Analyzing Table 7, it can be seen that the results obtained with meshes 1 (218,704 elements) and 2 (508,325 elements) showed significant variations, in some variables, with a maximum error of 13.19% reached for the average oil mass flow rate at the permeate exits. However, a comparison between the results obtained with meshes 2 (508,325 elements) and 3 (853,536 elements) shows little significant variations, that is, when compared with the analysis carried out between meshes 1 and 2, presenting a maximum error of 1.93% for the average mass flow rate at the permeate outlets.

**Figure 3:**
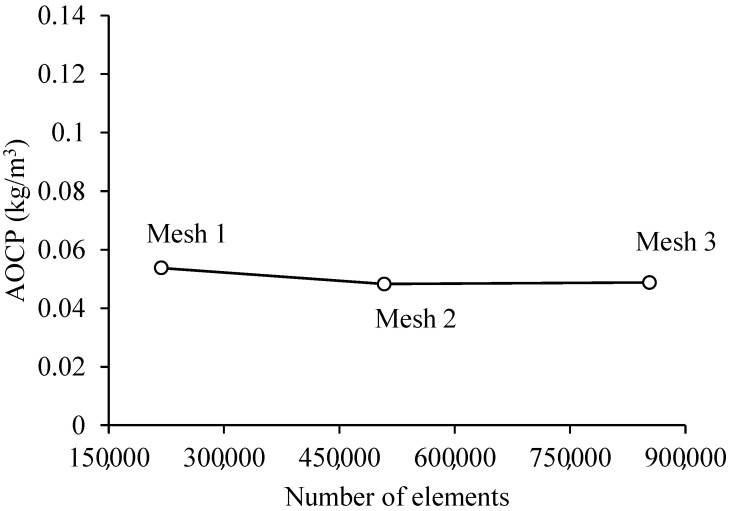
Average oil concentration in the permeate outlets for the three meshes used in the research (Cases 1, 2, and 3).

Thus, mesh 2 was chosen for the simulations, as it has an intermediate degree of refinement between the studied meshes and a reduced computational time. The greater the number of elements in the mesh, the greater the computational time. Besides, it was observed that the average oil volumetric concentration at the permeate outlets (Figure 3), for meshes 1, 2, and 3, showed very close results.

### 3.2. CFD Analysis

**Figure 4:**
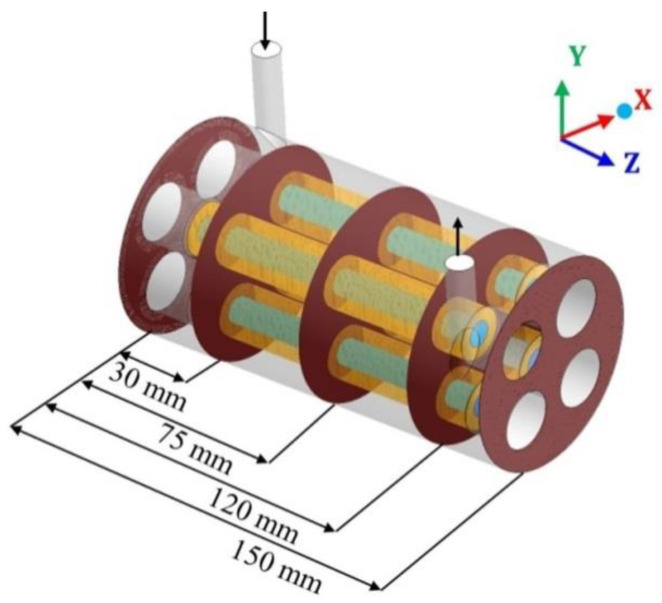
Transverse planes, in the hull region, chosen for data analysis.

To study the characteristics of the filtration process during CFD simulation for the case 2 (Table 6), different transversal planes were chosen for the module’s geometry as illustrated in Figure 4.

#### 3.2.1. Relative Volume Fraction of Oil in the Filtration Module

**Figure 5:**
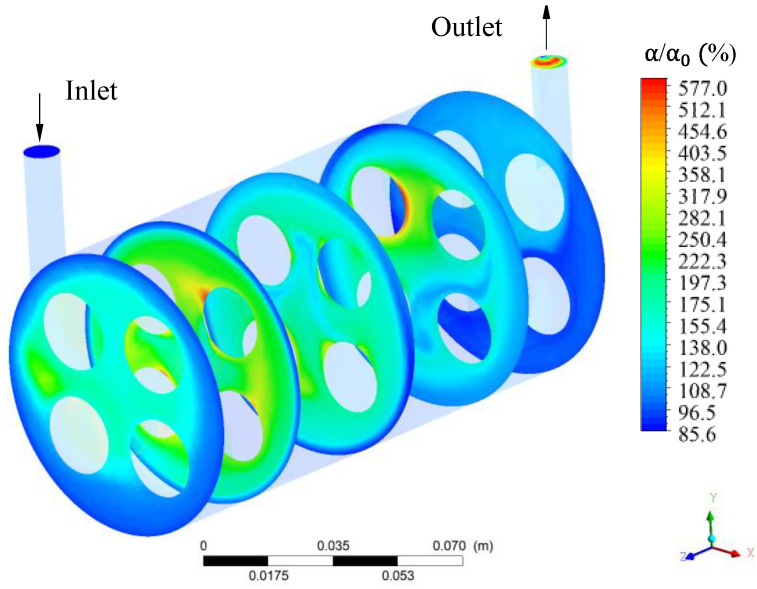
Relative oil volumetric fraction distribution at different plans along the hull of the filtration module during the water/oil separation process.

Figure 5 illustrates the distribution of the volumetric fraction inside the module. In this analysis, the volumetric fraction of oil in the fluid is compared to the volumetric fraction of oil in the feed mixture. Upon analyzing this figure, it can be seen that the oil volume fraction is minimal in the outermost region of the hull, indicating that the oil flows together with the feed mixture.

Considering the hull region as a whole, it is observed that the oil volumetric fraction varied from 85.6 to 577.0%. Exactly at the outlet of the hull (considering the xy plane when z = 150 mm), it is possible to observe a high volumetric fraction of oil at the location of the module’s outlet tube, indicating that the oil is separated from the water/oil mixture with high efficiency. Still in the plane of origin of the hull, it is possible to observe a lower oil concentration on the outer sides of the hull and higher oil concentration close to membrane surface indicating that the presence of oil in the hull is reduced, as the fluid goes towards the exit of the module. Furthermore, it can be seen that the oil distribuition is varied, in general, presenting higher values close to the inlet region of the membrane module and closest to membrane surface.

**Figure 6:**
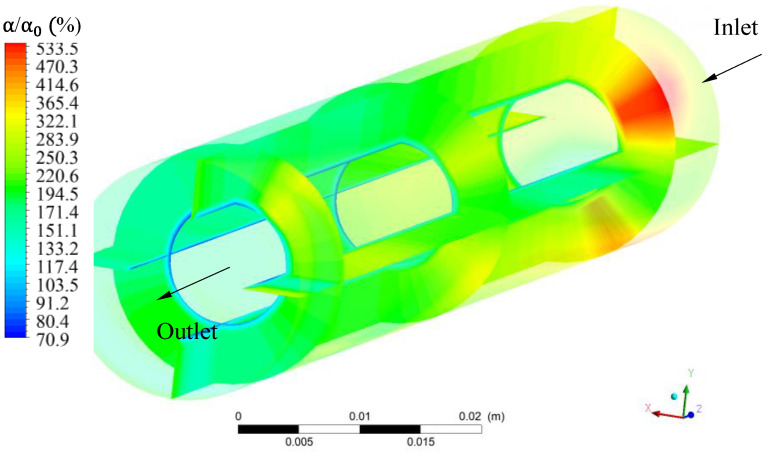
Relative oil volumetric fraction distribution at different regions of membrane 1 during the water/oil separation process.

When evaluating the relative oil volumetric fraction in the porous membranes (Figure 6), a great variation was observed inside the membranes. It can be seen that the relative volumetric fraction of oil varied from 70.9 to 533.5%. In addition, the region with the highest relative volumetric fraction of oil in membrane 1 was close to the inlet region of the module, while, other membranes have shown the lowest oil concentrations.

In general, the accumulation of relative oil fractions in the hull, membranes and permeate occurred due to the drag force and the tangential flow direction, which carries the retained particles to the terminal end of the module. Additional resistance to mass transfer occurs due to the establishment of a concentration gradient, leading to a decrease in the permeate flow. Therefore, this is a phenomenon that must be controlled and minimized, as it reduces the permeate flow and can affect the quality of the product.

#### 3.2.2. Pressure in the Filtration Module

**Figure 7:**
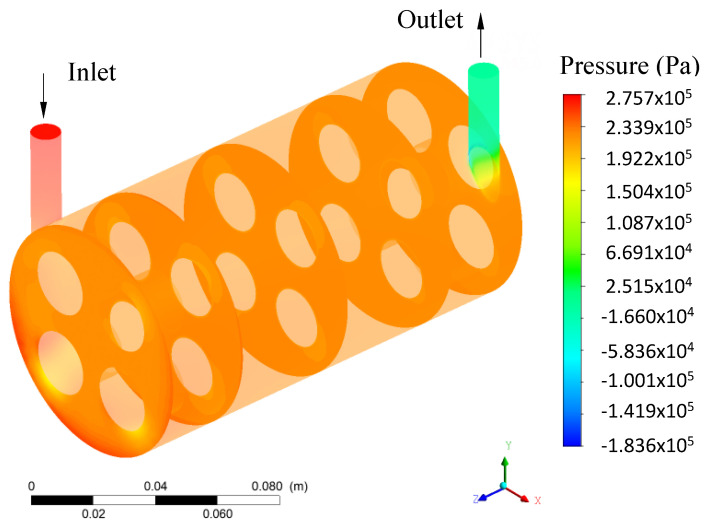
Pressure distribution in the hull region.

Figure 7 illustrates the pressure distribution in the hull region. Upon analyzing this figure, it can be observed that the pressure in the hull was higher in the region of entry and close to this region, reaching the lowest pressure values at the module outlet. A total pressure variation of 459.3 kPa was found. Observing the transverse planes at positions z = 0, 30, 75, 120, and 150 mm of the hull, it is noted that the pressure tends to be greater in the lower region of the hull, especially in the region below and between membranes 3 and 4.

**Figure 8:**
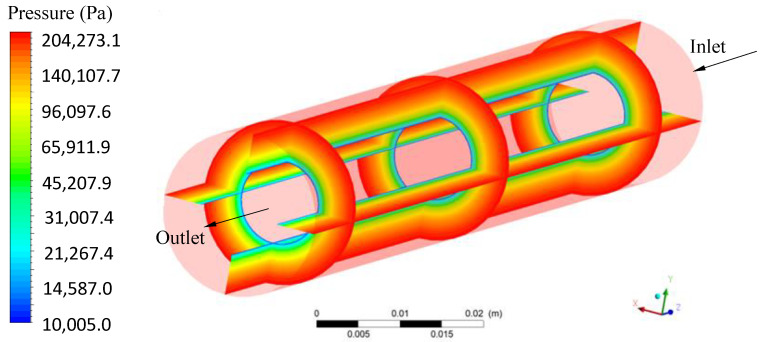
Pressure on the membrane at different, horizontal and vertical positions, of the filtration module.

The pressure along the membrane was assessed as illustrated in Figure 8. The pressure at the membrane surface was practically constant along the z axis (longitudinal length), observed by the similar values in the positions z = 30, 75 and 120 mm of the entrance, and varying in the x and y directions (radial direction). This behavior indicates an efficient system to maintain the same pressure throughout the filtration membrane module. In turn, the pressure differential in the system (transmembrane pressure) is a determining factor, to ensure the flow through the membrane, keeping the high filtration process efficiency for a longer period of time.

#### 3.2.3. Mixing Speed in the Filtration Module

**Figure 9:**
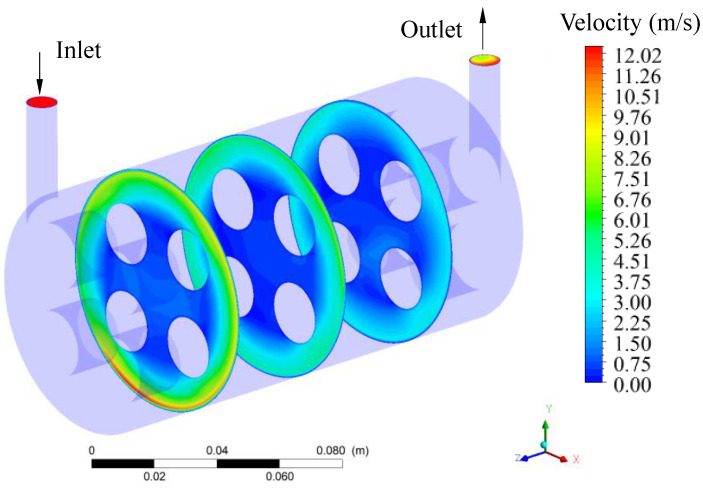
Velocity distribution of the fluid mixture inside the hull ot the filtration module during the water/oil separation process.

Figure 9 illustrates the velocity distribution of the mixture at different planes within the hull. Analyzing this figure, it can be seen that the velocity decreased in the direction of entry–exit and in the direction hull surface to membrane surface (radial direction). In fact, the velocity at the initial position of the membrane surface was almost null.

Finally, although CFD results have not be presented in detail, some results related to pressure, oil volume fraction and velocity distribution inside the hull and membrane of the module system used here, can be found in the reference [52].

### 3.3. Analysis of Hydrodynamic Parameters

#### 3.3.1. Effect of the Mass Flow of the Fluid Mixture at the Inlet of the Filtration Module

**Figure 10:**
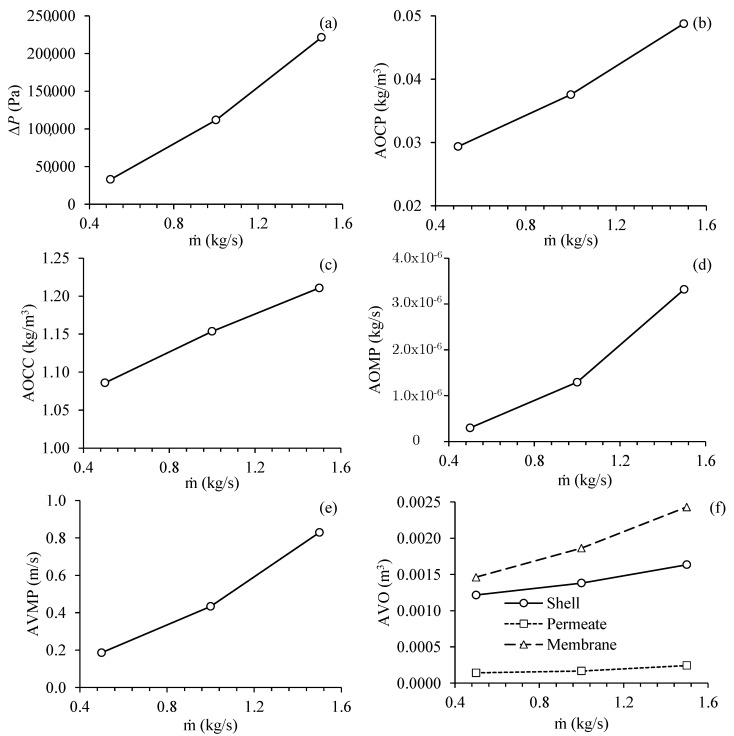
Process parameters in the filtration module as a function of the mass flow rate of fluid in the feed. (**a**) Transmembrane pressure, (**b**) average oil volumetric concentration at the permeate outlets, (**c**) average oil volumetric concentration at the concentrate outlet, (**d**) average oil flow rate at the permeate outlets, (**e**) average fluid velocity in the permeate outlets and (**f**) average volume of oil present in the hull, membranes and permeate (Cases 2, 4 and 5).

Figure 10 illustrates the results obtained for the different process parameters as a function of the fluid mass flow rate in the feed of the separation module, for Cases 2, 4, and 5. Analyzing this figure, it can be verified that the transmembrane pressure presents an approximately linear behavior with the mass flow rate of fluid in the feed, in the established operational range. Besides, it is observed that an increase in the mass flow rate of the mixture in the feed from 0.5 to 1.5 kg/s raised the transmembrane pressure from 33.00 to 221.32 kPa, which represents a percentage increase of 570.67%, in this process parameter. This increase is approximately 6.71 times and corresponds to an average increase of 2.592 times for each increase of 0.5 kg/s, within the studied range (Figure 10a). For a better understanding, the transmembrane pressure was obtained by the difference between the average pressure at the external surface of the membrane and average pressure at the internal surface (permeate duct).

The increase in the mass flow rate of the mixture in the feed also increased the average oil concentration at the permeate outlets (Figure 10b), the average oil concentration at the concentrate outlet (Figure 10c), and the average velocity of the fluid mixing at the permeate outlets (Figure 10e). It is important to mention a variation in the average oil volumetric concentration at the output of concentrate from 1.09 to 1.21 kg of oil per m^3^, while a variation in the average oil volumetric concentration at the outputs of the permeate from 0.029 to 0.049 kg of oil was obtained per m^3^, higher than that required by Brazilian legislation, which is 0.020 kg/m^3^ (20 mg/L) [5]. The average fluid velocity at the permeate outlets increased from 0.19 to 0.83 m/s, which represents an increase of 336.84%, for a variation in the mass flow rate of feed from 0.5 to 1.5 kg/s. This represents an increase in the average oil volumetric concentration at the permeate outputs of 68.96%, indicating that the system can be considered more efficient for lower values of feed mass flow rate in the studied operational range. Despite the increase in oil concentration at the permeate outlets, the effect on the permeate velocity was 4.37 times greater. Thus, the increase in the average oil volumetric flow at the permeate outlets (Figure 10d) can be attributed mainly to the increase in the flow velocity at the permeate outlets, which is due to the increase in transmembrane pressure.

Together, the high pressure on the inner surface of the membranes, favored mainly by the increase in the mass flow rate of feed, increased the pressure gradient between the outer and inner surfaces of the membranes (transmembrane pressure). As the filtration occurs preferably under a higher pressure gradient, where the hydraulic resistance is also lower [49], the increase in the mass flow rate of fluid in the feed favors a greater flow of filtrate, however it increases the polarization layer, mainly by raising the pressure in the system.

Analyzing Figure 10f, it can be seen that the greater the mass flow rate of fluid in the feed, the greater the volume of oil in the porous medium (membrane). The volume of oil in the hull also increased with the increase in the mass flow rate of fluid in the feed, however, to a lesser extent than that observed for the membranes. Finally, the volume of oil in the permeate was hardly affected by the increase in the mass flow rate of fluid in the feed, indicating that the membrane was able to retain part of the oil present in the feed fluid mixture as desired.

#### 3.3.2. Effect of Oil Concentration at the Entrance of the Filtration Module

**Figure 11:**
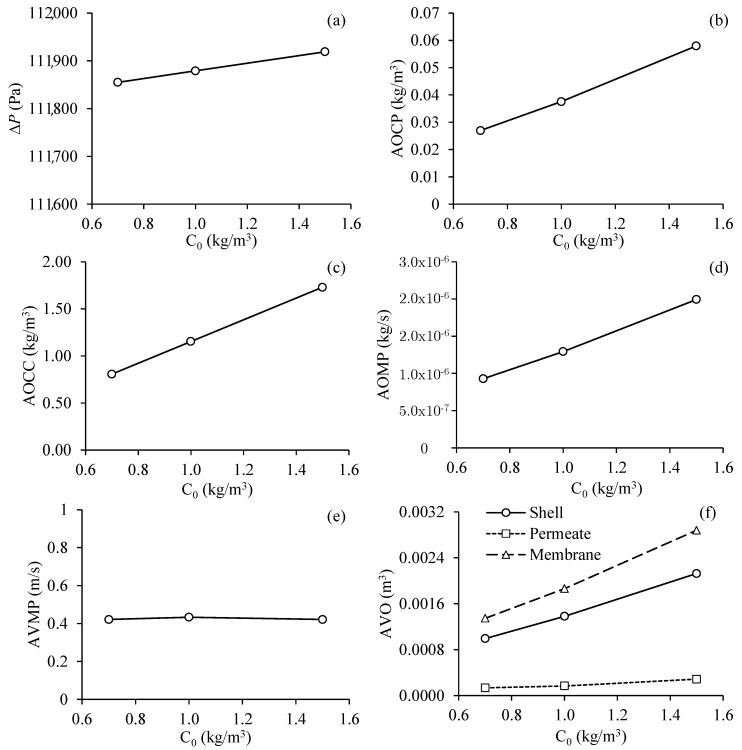
Process parameter in the filtration module as a function of the oil volumetric concentration in the feed. (**a**) Transmembrane pressure, (**b**) average oil volumetric concentration at the permeate outlets, (**c**) average oil volumetric concentration at the concentrate outlet, (**d**) average oil flow rate at the permeate outlets, (**e**) average fluid velocity in the permeate outlets and (**f**) average volume of oil present in the hull, membranes and permeate (Cases 5, 6 and 7).

Figure 11 illustrates the results obtained for the different process parameters as a function of the oil concentration at the inlet of the separation module, for Cases 5, 6, and 7. Analyzing Figure 11a, it can be seen a small effect of the oil concentration at the entrance of the system under transmembrane pressure, ranging from 111.86 to 111.92 kPa, which corresponds to a percentage increase of 0.05%. However, the higher the oil volumetric concentration at the inlet of the system, the greater the average oil volumetric concentration at the permeate outlets (Figure 11b), the greater the average oil volumetric concentration at the concentrate outlet (Figure 11c), and the greater the average oil mass flow rate at the permeate outlets (Figure 11d).

The oil concentration at the entrance of the system practically did not influence the average velocity of the mixture at the permeate exits, observing an average velocity of 0.425 m/s (Figure 11e). However, the higher oil volumetric concentration at the entrance of the system resulted in an increase in the volume of oil in the hull, in the membrane, and with less intensity in the permeate, as illustrated in Figure 11f.

In general, one can perceive a linearly approximate behavior of all hydrodynamic parameters as a function of the oil volumetric concentration at the entrance of the separation module.

#### 3.3.3. Effect of Oil Particle Diameter at the Entrance of the Filtration Module

**Figure 12:**
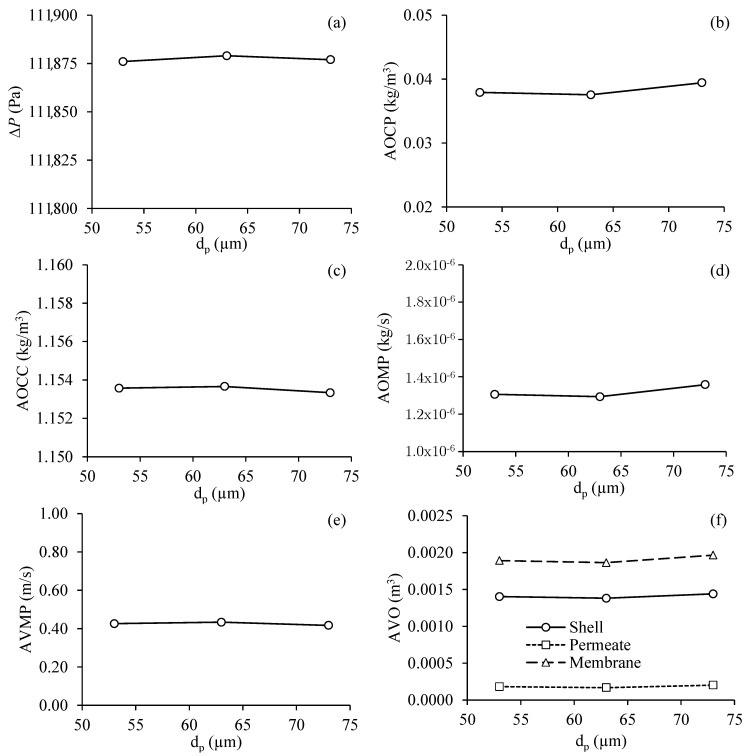
Process parameters in the filtration module as a function of the diameter of the oil drop. (**a**) Transmembrane pressure, (**b**) average oil volumetric concentration at the permeate outlets, (**c**) average oil volumetric concentration at the concentrate outlet, (**d**) average oil mass flow rate at the permeate outlets, (**e**) average fluid velocity in the permeate outlets and (**f**) average volume of oil present in the hull, membranes and permeate (Cases 5, 8 and 9).

Figure 12 shows the results obtained for the different process parameters as a function of the diameter of the oil dropat the inlet of the separation module, for Cases 5, 8, and 9. From an analysis of Figure 12, it appears that the diameter of the oil particles hardly influenced the parameters of the filtration process. It was observed that the transmembrane pressure, the average oil volumetric concentration in the permeate and concentrate outlets, the average oil mass flow rate in the permeate outlets, the average velocity of the mixing in the permeate outlets, and the average volume of oil present in the hull, in the membranes and the permeate, remained practically constant for different diameters of the oil drop. This is mainly due to the membrane parameters, dimensions, shape, permeability, and porosity, which are high for this diameter range of the oil drop.

#### 3.3.4. Effect of Membrane Permeability of the Filtration Module

**Figure 13:**
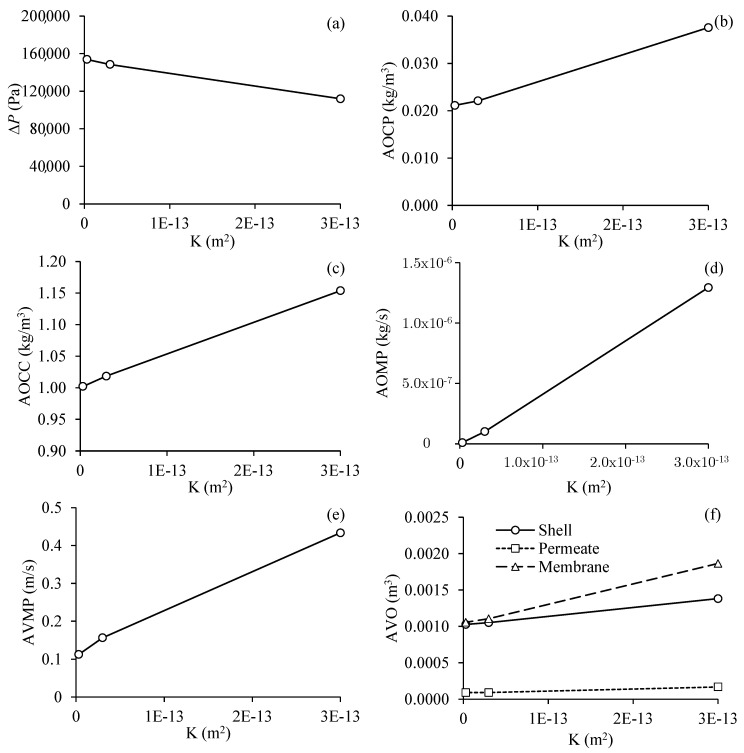
Process parameter in the filtration module as a function of membrane permeability. (**a**) Transmembrane pressure, (**b**) average oil volumetric concentration at the permeate outlets, (**c**) average oil volumetric concentration at the concentrate outlet, (**d**) average oil mass flow rate at the permeate outlets, (**e**) average velocity of the fluid mixture at the permeate outlets and (**f**) average volume of oil present in the hull, membranes and permeate (Cases 5, 10 and 11).

Figure 13 shows the results obtained for the different process parameters as a function of the permeability of the membranes, for Cases 5, 10, and 11. From an analysis of this figure, it can be seen that an increase in membrane permeability resulted in lower pressure on the filtration system (Figure 13a). The increase in membrane permeability from 3 × 10^−15^ to 3 × 10^−13^ m^2^ reduced the transmembrane pressure from 153.82 to 111.88 kPa, which corresponds to a reduction of 22.77%. On the other hand, the increase in the permeability of the membranes resulted in a higher average oil volumetric concentration at the permeate exits and the concentrate outlet, greater average oil mass flow rate at the permeate exits, as well as a higher average velocity of the fluid mixing at the permeate exits.

A high permeability between the porous layers is the key to obtain a good flow distribution during membrane filtration and to limit the fouling phenomenon [49], favoring a higher filtration rate. The higher the permeability of the membranes, the higher the average fluid velocity at the permeate outlets (Figure 13e) and the higher average oil volumetric concentration at the concentrate outlets (Figure 13c), however, caused a higher average oil volumetric concentration at the permeate outlets (Figure 13b), which is not desirable.

It can be considered that the increase in the average oil volumetric concentration at the permeate outlets was mainly due to the decrease in filtration efficiency. The higher the membrane permeability, the greater the average flow of oil in the permeate outlets, demonstrating that, in addition to the increase in permeability favoring a higher average speed in the permeate outlets, the oil mas flow rate in the permeates also increased, indicating less filtration potential and poor quality of filtered water, but there was a significant increase in the average velocity of the fluid at the permeate outlets.

Finally, the increase in the permeability of the membranes also increased the volume of oil present in the membranes, which indicates the greater separation of the water/oil mixture (Figure 13f), slightly affected the volume of oil present in the hull and hardly influenced the volume of oil present in the permeate, but practically doubled the oil volumetric concentration in the permeate.

#### 3.3.5. Effect of Membrane Porosity of the Filtration Module

**Figure 14:**
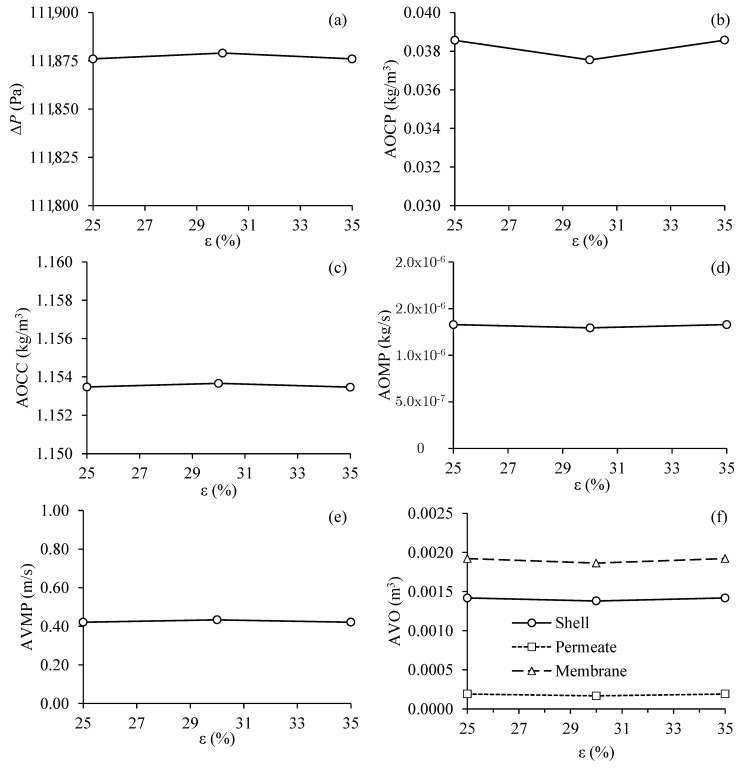
Process parameter in the filtration module as a function of the porosity of the membrane. (**a**) Transmembrane pressure, (**b**) average oil volumetric concentration at the permeate outlets, (**c**) average oil volumetric concentration at the concentrate outlet, (**d**) average oil mass flow rate at the permeate outlets, (**e**) average velocity of the fluid mixture at the permeate outlets and (**f**) average volume of oil present in the hull, membranes and permeate (Cases 5, 12 and 13).

Figure 14 shows the results obtained for the different process parameters as a function of the porosity of the separation module membranes, for Cases 5, 12, and 13. Analyzing the results plotted in Figure 14, it can be seen that an increase in the porosity of the membranes from 25 to 35%, practically did not influence the transmembrane pressure, the average oil volumetric concentration in the permeate and concentrate outlets, the average oil mass flow rate in the permeate outlets, the average velocity of the fluid mixture at the permeate outlets and the average volume of oil present in the hull, membranes and permeate, within the established operational range.

Alves et al. [51] studied the water/oil separation process numerically, using a double-tube type module and with the tangential flow in a ceramic membrane system. Unlike what was observed in this work, these authors reported that porosity influences filtration properties. According to the authors, the lower the porosity, the greater the velocity gradient in the annular space, the larger the membrane area that contains recirculation zones, and the greater the separation efficiency. However, it is noteworthy that the highest percentage of porosity applied in this work is equal to the lowest percentage studied by Alves et al. [51], who applied porosity ranging from 35 to 44%.It is important to notice that the model used in this paper is essentially macroscopic, and pore size and non-uniform distribution are not considered.

### 3.4. Perfomance Analysis of Membrane Module

The membrane performance for water–oil separation was analyzed for each case studied. The calculation of the separation efficiency (in terms of the rejection coefficient) was performed as follows:

(18) $$R=1-\;\left(\frac{{\dot{m}}_{\mathrm{outlet}}}{{\dot{m}}_{\mathrm{inlet}}}\right)$$

where ${\dot{m}}_{\mathrm{outlet}}$ represents total oil mass flow rate at the permeate, and ${\dot{m}}_{\mathrm{inlet}}$ is the oil mass flow rate at the feed inlet.

Table 8 summarizes the mass flow rate of the oil and water in the inlet and outlet (permeate and concentrate) of the module, and separation efficiency of the device for each studied case. Upon analyzing this table, it is verified that mass conservation was obtained in each case. The rejection coefficient ranged from 0.9991 ≤ R ≤ 0.9999. For the condition of a higher rejection coefficient, reduction in the permeate flow and increases in the resistance of the fluid to flow through the pores of the membrane were verified. As a consequence, there is smaller amount of oil transported by convection through the porous membrane and, thus, reducing in the oil concentration at the permeate outlet, and increasing separation efficiency.

## 4. Conclusions

This research evaluated the behavior of different process parameters in a porous ceramic membrane module used in the treatment of oily water. For this purpose, the computational fluid dynamics technique proved to be an efficient and powerful tool in the analysis of this physical phenomenon. From the results obtained, it can be concluded that an increase in the inlet mass flow rate from 0.5 to 1.5 kg/s, caused an increase in transmembrane pressure from 33 to 221.32 kPa. The high-pressure gradient between the external and internal surfaces of the membrane affected the filtration flow, observed by the increase in the average velocity of the fluid in the permeate outlets from 0.19 to 0.83 m/s, an increase in the oil volumetric concentration in the permeate outlets from 1.09 to 1.21 kg/m^3^, and lower average oil mass flow rate at the permeate outlets. A variation in the diameter of the oil drop (53 to 73 µm) and the porosity of the membranes (25 to 35%) did not significantly influence the filtration parameters of the module: transmembrane pressure, average oil volumetric concentration in the permeate outlets, average oil volumetric concentration at the concentrate outlet, average oil mass flow rate at the permeate outlets, the average velocity of the fluid mixture at the permeate outlets, average oil volume in the hull, average oil volume in the permeate and average oil volume in the membranes. In turn, with an increase in membrane permeability from 3 × 10^−15^ to 3 × 10^−13^ m^2^, transmembrane pressure was reduced from 153.82 to 111.88 kPa (22.77% reduction), the average oil volumetric concentration at the permeate outlets increased from 0.021 to 0.037 kg/m^3^ and at the concentrate outlet from 1.00 to 1.154 kg/m^3^; the average oil mass flow rate and the average velocity of the fluid at the permeate outputs also increased.

## Figures and Tables

**Table 1:** Geometric dimensions of the shell-tube separation module.

Geometric Parameter	Dimension (mm)
Separator module length	150
Useful length of each membrane	114
Length of each membrane support	18
The diameter of the separation module	80
The inner diameter of each membrane	10
The outer diameter of each membrane	20
The outside diameter of each support	22
Inlet duct diameter	10
Outlet duct diameter	10
Inlet duct length	60
Outlet duct length	60
Membrane thickness	10

**Table 2:** Boundary conditions used in the simulations.

Region	Boundary Condition
Tab entry	Prescribed mass flow
Separator outputs (Permeate 1, 2, 3, and 4 and concentrated)	Prescribed pressure
Internal and external surfaces of membranes	Interior (available in Fluent software)
Internal hull surfaces	Wall with zero speed
Surfaces surfaces	Wall with zero speed
Membrane ends (hull and permeate)	Wall with zero speed

**Table 3:** Ansys Fluent standard relaxation factors.

Relaxation Factors	
Density	1.00
Body forces	1.00
Turbulent kinetic energy	0.80
Turbulence dissipation rate	0.80
Turbulent viscosity	1.00
Volumetric fraction	0.50
Linear momentum	0.75
Pressure	0.75
Courant number	200.00

Source: [61].

**Table 4:** Convergence criteria used.

Equation	Convergence Criterion
Linear momentum in x, y, and z directions	0.001
Continuity	0.001
k	0.001
ω	0.001
Volumetric fraction	0.001

Source: [61].

**Table 5:** Thermo-physical properties of oil and water.

Physical Properties	Oil	Water
ρ (kg/m^3^)	997.00	998.20
μ (Pa.s)	0.05	0.001003

Source: [39].

**Table 6:** Parameter data used in the simulations [39].

Case	Mixture			Membrane		Mesh
	$\dot{m}\;(kg/s)$	$C_{0}(\mathrm{kg}/m^{3})$	$d_{p\;}\left(\mu m\right)$	$K\;\left(m^{2}\right)$	ε	
1	1.5	1.0	63	3 × 10^−13^	30	1
2	1.5	1.0	63	3 × 10^−13^	30	2
3	1.5	1.0	63	3 × 10^−13^	30	3
4	0.5	1.0	63	3 × 10^−13^	30	2
5	1.0	1.0	63	3 × 10^−13^	30	2
6	1.0	1.5	3	3 × 10^−13^	30	2
7	1.0	0.7	63	3 × 10^−13^	30	2
8	1.0	1.0	53	3 × 10^−13^	30	2
9	1.0	1.0	73	3 × 10^−13^	30	2
10	1.0	1.0	63	3 × 10^−14^	30	2
11	1.0	1.0	63	3 × 10^−15^	30	2
12	1.0	1.0	63	3 × 10^−13^	35	2
13	1.0	1.0	63	3 × 10^−13^	25	2

**Table 7:** Operational parameters used in the mesh study.

Mesh	ΔP (Pa)	AOCP (kg/m^3^)	AOCC (kg/m^3^)	AOMP (kg/s)	AVMP (m/s)	AVOH (m^3^)	AVOP (m^3^)	AVOM (m^3^)
**1**	227.236	0.0537395	1.216620	3.80 × 10^−6^	0.848601	0.001658	0.00022	0.002674
**2**	221.322	0.0487558	0.210770	3.30 × 10^−6^	0.829478	0.0016340	0.00024	0.002431
**3**	218.739	0.0483874	1.207950	3.25 × 10^−6^	0.815305	0.0016381	0.00023	0.002403
**Error_12_**	2.67%	10.22%	0.48%	13.19%	2.31%	1.45%	2.31%	9.95%
**Error_23_**	1.18%	0.76%	0.23%	1.93%	1.74%	0.25%	2.31%	1.17%

**Table 8:** Mass flow rate at the inlet and outlet of the module system and rejection coefficient for all simulated cases.

Case	Mass Flow Rate (kg/s)						R (%)
	Inlet		Permeate Outlet		Concentrate Outlet		
	Oil	Water	Oil	Water	Oil	Water	
**2**	0.001503	1.4985	13.000 × 10^−6^	2.690 × 10^−1^	0.001490	1.229770	0.9913
**4**	0.001002	0.9990	5.170 × 10^−6^	1.370 × 10^−1^	0.000997	0.861508	0.9948
**5**	0.001503	1.4985	1.330 × 10^−6^	2.720 × 10^−1^	0.001490	1.22667	0.9911
**6**	0.001503	0.9985	7.980 × 10^−6^	1.370 × 10^−1^	0.001495	0.861098	0.9947
**7**	0.000701	0.9993	3.720 × 10^−6^	1.380 × 10^−1^	0.000697	0.861765	0.9947
**8**	0.001002	0.9990	5.220 × 10^−6^	1.370 × 10^−1^	0.000997	0.861514	0.9948
**9**	0.001002	0.9990	5.430 × 10^−6^	1.370 × 10^−1^	0.000997	0.861514	0.9945
**10**	0.001002	0.9990	0.400 × 10^−6^	0.183 × 10^−1^	0.001002	0.980746	0.9996
**11**	0.001002	0.9990	0.040 × 10^−6^	0.019 × 10^−1^	0.001002	0.997109	0.9999
**12**	0.001002	0.9990	5.310 × 10^−6^	1.370 × 10^−1^	0.000997	0.861519	0.9947
**13**	0.001002	0.9990	5.310 × 10^−6^	1.370 × 10^−1^	0.000997	0.861519	0.9947
